# Synthesis and Self-Organization of Fluorene-Conjugated Bisimidazolylporphyrin and Its Optical Properties

**DOI:** 10.3390/ijms14010322

**Published:** 2012-12-21

**Authors:** Kazuya Ogawa, Naoyuki Makiuchi, Yoshiaki Kobuke

**Affiliations:** Graduate School of Materials Science, Nara Institute of Science and Technology, 8916-5 Takayama, Ikoma, Nara 630-0192, Japan; E-Mail: ma_naoyu@yahoo.co.jp (N.M.)

**Keywords:** porphyrin, self-assembly, two-photon absorption, fluorene, imidazole, Z-scan

## Abstract

A conjugated-bisimidazolylporphyrin bridged by bis(ethynylfluorene) was synthesized and organized into linear polymer through self-coordination having mean molecular weights, *M*_w_ and *M*_n_, of ~2.1 × 10^5^ Da and ~1.6 × 10^5^ Da, respectively. A large two-photon absorption cross section value of 3.4 × 10^5^ GM (per dimer unit) was observed. This value was comparable to that of the previously reported self-assembled linear polymer consisting of butadiyne-bridged imidazolylporphyrins. The two-photon absorption properties could be controlled by tuning the wavelength and absorption intensity of the one-photon absorption.

## 1. Introduction

Recently, much attention has been focused on organic dye molecules in view of optical applications such as organic solar cell [1], electronic luminescence [2], photon-mode optical memory using photochromic molecules [3], and nonlinear optics including two-photon absorption (2PA) materials [4–11]. Novel functions would be expected upon conjugation of two or more chromophores, even when a monomeric dye itself exhibits insignificant function. We developed two-photon absorption materials [12–22] using self-assembled multi-porphyrin arrays and found that the extension of the π-conjugation between the porphyrins with triple bonds was the most significant for 2PA enhancement. Complementary coordination of imidazolyl to zinc, in which the stability constant of the complementary dimer exceeded 10^11^ M^−1^ in CHCl_3_ [23–25], also contributed to the enhancement. However, an additional expansion of π-conjugation gave rise to a large red-shift of the S_0_→S_1_ one-photon absorption (1PA) to the two-photon resonance range (800 nm to 900 nm) [22]. To prevent such an overlap of 2PA and 1PA, it is necessary to control the position of the S_0_→S_1_ one-photon absorption by tuning the interaction between chromophores. Fluorene is a well-known π-spacer for two-photon absorption materials [26–33] and provides an angle of 160° between two ethynylimidazolylporphyrins when these are connected at the 2- and 7-positions of fluorene (**1**). Therien and Anderson reported bisporphyrins bridged by a π-spacer comprising an acetylenic connection [5,34]. Compound **1** gave the linear polymer **1P** by zinc-imidazolyl complementary coordination. Polymer **1P** could easily be dissociated into the unit **1M** by ligand coordination, such as with pyridine and imidazole. The slightly bent angle of 160° was expected to reduce the exciton interaction between the two porphyrins and shift the 1PA bands to shorter wavelengths than those of the butadiyne-bridged bisimidazolylporphyrin array **2P** without fluorene, allowing the measurement of the hidden 2PA bands in the shorter wavelength range. Similar fluorene-conjugated bisimidazolylporphyrins having two allyl groups at *meso*-positions have been synthesized to construct large macrocyclic arrays. The self-coordinated structures in the macrocycles were fixed by the ring-closing metathesis reaction of the allyl groups to investigate the effect of cyclization on 2PA by a femtosecond fluorescence method in the range between 800 and 875 nm [20]. Here, we report the synthesis of linear polymers self-organized by fluorene-conjugated bisimidazolylporphyrins without allyl groups, and their 2PA properties in the range from 740 to 930 nm.

## 2. Results and Discussion

### 2.1. Synthesis

**Figure 1:**
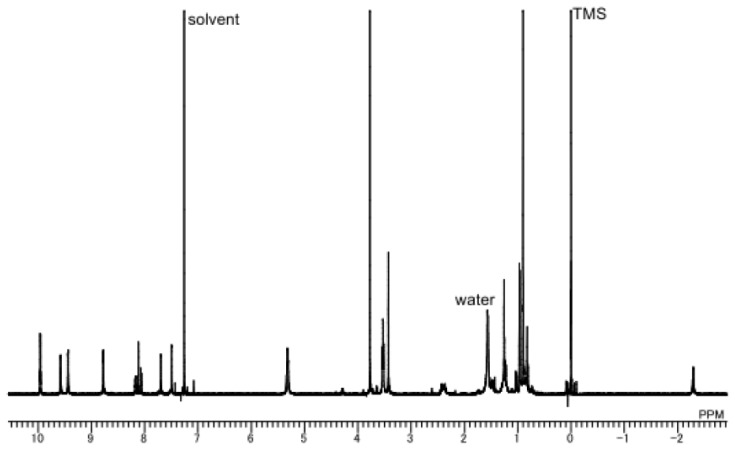
600 MHz ^1^H NMR spectrum of free base **5** in CDCl_3_.

**Scheme 1:**
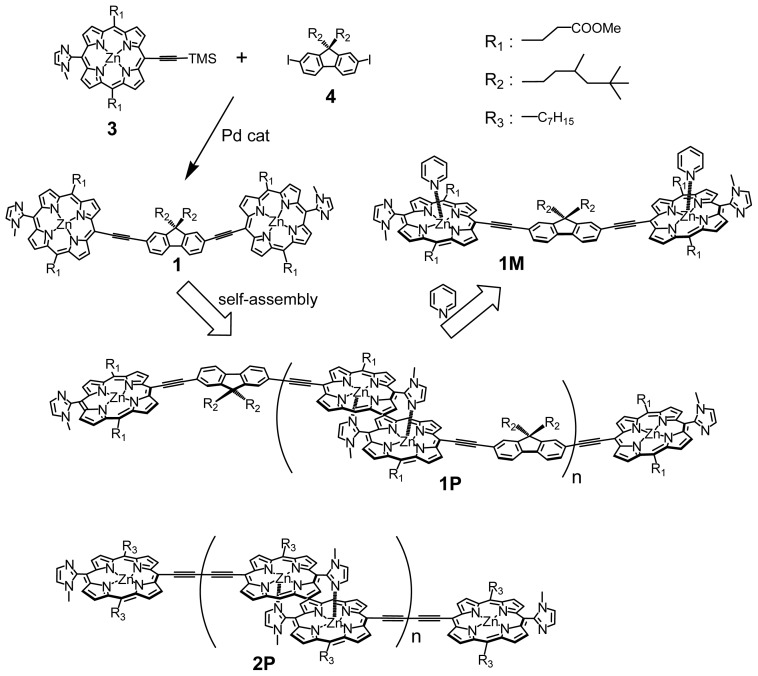
Self-assembled imidazolylporphyrins connected by triple bond.

Bisporphyrin **1** was synthesized from 5,15-bis(2-methoxycarbonylethyl)-10- (trimethylsilylpropargyl)-20-(1-methyl-2-imidazolyl)porphyrinatozinc **3** [15] and 2,7-diiodo-9,9- bis(3,5,5-trimethylhexyl)fluorene **4** [35,36] using a Pd_2_(dba)_3_/AsPh_3_ catalytic system, where deprotection of the TMS group and the coupling reaction were conducted in a one-pot procedure because of the low solubility of the deprotected ethynylimidazolylporphyrin (Scheme 1). Purification by preparative gel permeation chromatography (GPC) with pyridine elution allowed for the isolation of **1** from the crude mixture in 33% yield. Both the MALDI-TOF mass spectral and analytical GPC measurements showed a single species corresponding to the molecular weight of **1**. However, broad signals were obtained in the NMR spectrum of **1**, even in a coordinating solvent such as pyridine-*d*_5_. Accordingly, the free base **5** was prepared by treatment with HCl to obtain a clearer NMR spectrum (Figure 1). Bisporphyrin **1** could not be obtained when 2,7-dibromofluorene was used as a starting material, due to its low reactivity.

### 2.2. Formation of Self-Assembled Polymer

**Figure 2:**
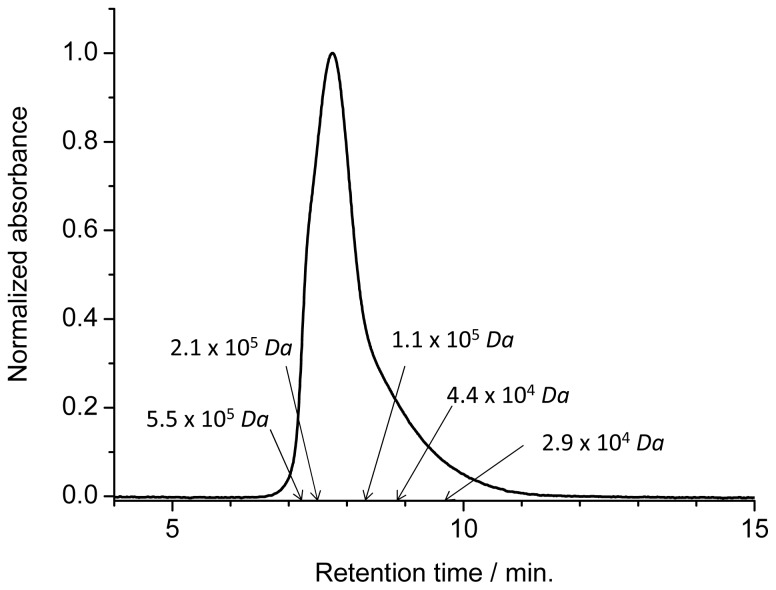
GPC chart of **1P** along with the peak positions of polystyrene standards.

When **1** was dissolved in a nonpolar solvent such as chloroform or 1,1,2,2,-tetrachloroethane after the evaporation of pyridine, the long linear polymer **1P** was formed by self-coordination. Polymer **1P** changed gradually to cyclic arrays of various sizes in chloroform above 45 °C or in chloroform/methanol (7:3, *v*/*v*%) at room temperature. Such cyclic arrays have been reported elsewhere [20]. **1P** retains its linear polymer structure in chloroform or in tetrachloroethane without methanol at room temperature sufficiently long for 2PA measurements. The molecular weight of **1P** was analyzed by GPC with chloroform elution on a column with an exclusion limit of 5 × 10^5^ Da. Figure 2 shows the GPC chart of **1P** monitored at 420 nm by an UV-vis photodiode array. The arrows indicate the peak positions of polystyrene standards. The absorption spectra of **1P** at different retention times between 7 and 10 min were identical. The distribution maximum of the elution curve of **1P** appeared around Mw 1.5 × 10^5^ Da. The mean molecular weights, *M*_w_ and *M*_n_, estimated by comparing the data with those of polystyrene standards [37], were ~2.1 × 10^5^ Da and ~1.6 × 10^5^ Da, respectively, which results in a polydispersity index of *M*_w_/*M*_n_ = 1.4. The mean molecular weight of *M*_n_ = 1.6 × 10^5^ corresponded to ca. 94 units of **1**, and these values were comparable with linear self-assembled bisporphyrin arrays [14].

### 2.3. UV-Vis Absorption Spectra

**Figure 3:**
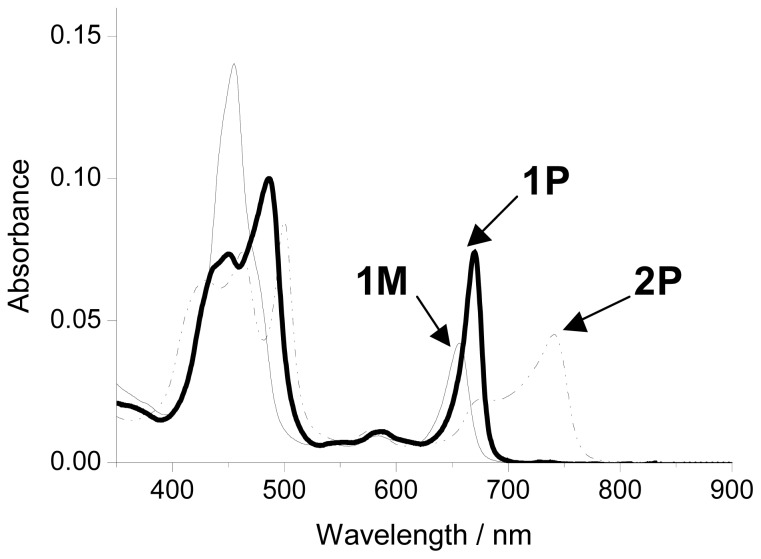
One-photon absorption spectra of **1P** (bold line) in tetrachloroethane, **1M** (thin line) in tetrachloroethane/pyridine (7:3, *v*/*v*), and **2P** (dashed line) in CHCl_3_.

The UV*-*vis absorption spectra of **1P** in tetrachloroethane (solid line), **1M** in tetrachloroethane/pyridine (7:3, *v*/*v*%), and **2P** in chloroform (dashed line) are shown in Figure 3. The Soret and Q-bands of **1P** were observed at around 485 and 670 nm, respectively. These were blue-shifted by 15 and 70 nm compared with those of **2P** (500 nm and 740 nm), suggesting a decrease in interaction due to the long distance and torsional angle of 160° between the two porphyrins. However, the absorption intensity of the Q-band in **1P** was still strong. On the other hand, the Q-band of unit **1M** was blue-shifted to 655 nm and its intensity was considerably decreased because of the disappearance of excitonic interactions through the zinc-imidazolyl complementary coordination.

### 2.4. Nonlinear Absorption

The 2PA cross sections of **1P** and **1M** were measured with an open-aperture Z-scan technique at wavelengths from 740 to 920 nm, using a Nd:YAG nanosecond pulse laser system with optical parametric oscillator. The nanosecond measurement system and analysis method were previously reported [14]. In general, the effective cross section value, ^eff^σ^(2)^, measured using nanosecond pulses, is 2–3 orders of magnitude larger than the pure 2PA cross section, σ ^(2)^, obtained with femtosecond pulses, because the excited state absorption (ESA) contributes to the data. In our porphyrin systems, the ^eff^σ ^(2)^ values were 30 to 50 times larger than the σ ^(2)^ values, and the 2PA spectra measured with nanosecond and femtosecond pulses were similar. Therefore, ^eff^σ ^(2)^ values obtained by nanosecond measurements were a good measure for the 2PA properties. Moreover, in some 2PA applications, such as 2PA-PDT and 3-D optical memory, nanosecond pulses will be more appropriate because of the ease of operation and availability.

**Figure 4:**
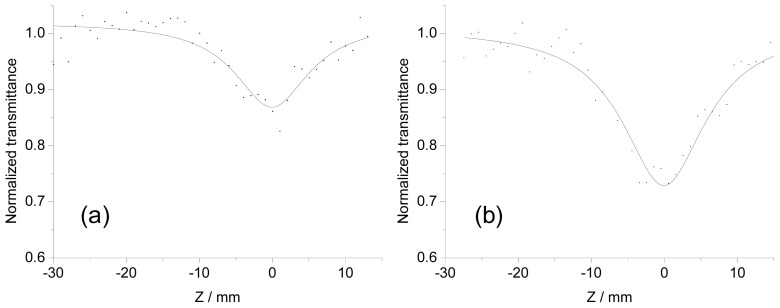
Typical open-aperture Z-scan traces for **1P** (dotted trace) and theoretically fitted curves (solid line) at 890 nm (**a** at a concentration of 7.66 × 10^−5^ M in 1,1,2,2,-tetrachloroethane with a pulse energy of 1.6 mJ) and 760 nm (**b** at a concentration of 6.87 × 10^−5^ M in 1,1,2,2,-tetrachloroethane with a pulse energy of 1.45 mJ).

**Figure 5:**
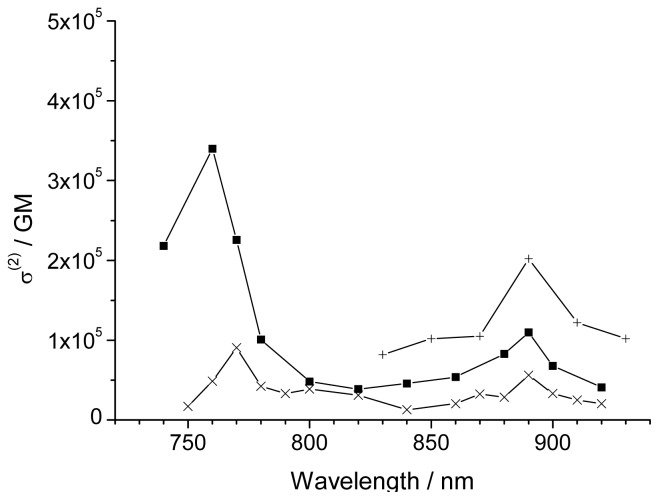
2PA spectra of **1P** (■), **1M** (×), and **2P** (+).

Figure 4 shows typical Z-scan traces for **1P** in tetrachloroethane (dotted trace) and theoretically fitted curves (solid line) at 890 nm ((a) at a concentration of 7.66 × 10^−5^ M with a pulse energy of 1.6 mJ) and 760 nm ((b) at a concentration of 6.87 × 10^−5^ M with a pulse energy of 1.45 mJ). The ^eff^σ ^(2)^ values were obtained from the fitted curves and theoretical equations [14,38]. Figure 5 shows 2PA spectra of **1P** (square). Two peaks were observed at 760 and 890 nm with large ^eff^σ ^(2)^ values of 3.4 × 10^5^ and 1.1 × 10^5^ GM (per dimer unit), respectively. 2PA spectra of **1M** (Figure 5, cross) also showed two peaks at 770 and 890 nm, with values of 9.1 × 10^4^ and 5.6 × 10^4^ GM, respectively. The values obtained for **1M** were almost one-third to one-half of those of **1P**. As described for the absorption spectra, **1P** showed strong one-photon absorption of the Q-band at 670 nm. On the other hand, that of **1M** was blue-shifted to 655 nm and the oscillator strength was almost half that of **1P**, indicating that the resonance enhancement for **1M**, which comes from the one-photon absorption near the two-photon resonance [38,39], would be expected to be poorer than that for **1P**. This may clearly explain the difference in the ^eff^σ ^(2)^ values between **1P** and **1M**, and we can confirm again that self-organization through complementary coordination is effective for the enhancement [13,14]. The shapes of the 2PA spectra of **1P** and **1M** were similar, but did not correspond to those of the one-photon spectra in the wavelength range of half of 2PA, *i.e.*, 370 nm to 500 nm, suggesting that the final state by 2PA is different from the one-photon excited state [5,39]. The ^eff^σ ^(2)^ values per dimer unit of **1P** in the wavelength range between 800 and 860 nm ranged from 3.9 × 10^4^ to 5.4 × 10^4^ GM. These values were similar to those of macrocycles measured by femtosecond pulses [20], indicating negligible ESA contributions in this wavelength range, considering nanosecond measurement.

In the previous report, the linear polymer **2P** exhibited a large ^eff^σ ^(2)^ value of 2.0 × 10^5^ GM (per dimer unit) at 890 nm [14], which was twice of that of **1P** at the same wavelength. **2P** had a strong one-photon absorption of the Q-band at 740 nm that was longer than **1P** by 70 nm, suggesting that **2P** undergoes a stronger resonance enhancement effect than **1P**. However, the shift of the strong Q-band to the shorter wavelength of 670 nm for **1P** allows the Z-scan measurements in the wavelength range of 720 to 820 nm, which is prohibited by one-photon absorption in the case of **2P**. Then, we could find another strong 2PA band at around 760 nm with the largest ^eff^σ^(2)^ value of 3.4 × 10^5^ GM. The cross sections increased with the shortening of the incident light wavelength below around 800 nm. Similar behavior was observed in simple porphyrins such as tetraphenylporphyrin, Zn octaethylporphyrin, and a self-assembled dimer measured using a femtosecond fluorescence method [39] or nanosecond Z-scan method [16]. This has been explained as a resonance enhancement near the one-photon absorption of the Q-band.

## 3. Experimental Section

### General

^1^H NMR spectra were obtained in CDCl_3_ with Me_4_Si as the internal standard (δ 0 ppm) and recorded on either a JEOL JNM EX270 or JEOL ECP600. UV-vis spectra were obtained on either a Shimadzu UV-1650PC or UV-3100PC. MALDI-TOF mass spectra were obtained on Perseptive Biosystems Voyager DE-STR and Shimadzu/KRATOS Axima-CFR Kompact MALDI with dithranol (Aldrich, St. Louis, MO, USA) or 2-[3-(4-*tert*-butylphenyl)-2-methylprop-2-enylidene]-malononitrile (DCTB, Aldrich) as the matrix. Reactions were monitored on silica gel 60 F254 TLC plates (Merck, Darmstadt, Germany). The silica gel utilized for column chromatography was purchased from Kanto Chemical Co. Inc.: Silica Gel 60N (Spherical, Neutral) 60–210 mm and 40–50 mm (Flash). The alumina used for column chromatography was purchased from Merck: Aluminum oxide 90 active basic. Analytical GPC measurements were performed on a HEWLETT PACKARD 1100 SERIES with a JAIGEL 3H-A or 4H-A column for chloroform eluent. The nanosecond Z-scan measurement system and analysis method were previously reported [14].

Synthesis of **1**: Under argon atmosphere, **3** [15] (95 mg, 132 μmol) and **4** [35,36] (35.4 mg, 52.6 μmol) were dissolved in dry THF (45 mL). Then, tetrabutylammonium fluoride in THF (264 μL, 264 μmol) was added. After 30 min, triethylamine (9 mL), Pd_2_(dba)_3_ (27.2 mg, 26.3 μmol), and AsPh_3_ (32.2 mg, 105 μmol) were added. After stirring 4 h, the reaction solvents were evaporated. The residue was dissolved in chloroform and washed with water. The crude residue after concentration was purified by preparative GPC (Tosoh G2500H_HR_, pyridine elution) to give **1** (29 mg, 33%). MALDI-TOF mass (dithranol) *m*/*z* 1711.66 (M + H)^+^, calcd for C_99_H_98_N_12_O_8_Zn_2_, 1710.62; UV-vis λ_max_ (nm, in CHCl_3_) 449, 484, 583, 667; fluorescence λ_max_ (nm, λ_ex_ = 484 nm, in CHCl_3_) 670, 731.

Synthesis of **5**: Under an Ar atmosphere, **1** (4.5 mg, 2.63 μmol) in chloroform (30 mL) was added into a mixture of 12 N hydrochloric acid (8 mL) and methanol (40 mL). After stirring for 30 min at room temperature, the reaction solution was diluted with water and extracted with chloroform. The chloroform layer was washed with saturated NaHCO_3_ aqueous solution. The crude residue after concentration was purified with silica gel column chromatography (CHCl_3_/MeOH = 20/1) (yield: 53%). ^1^H NMR (600 MHz, CDCl_3_) δ 9.96 (q, 4H, *J* = 4.8 Hz, β), 9.58 (q, 4H, *J* = 4.8 Hz, β), 9.44 (d, 4H, *J* = 4.8 Hz, β), 8.78 (d, 4H, *J* = 4.8 Hz, β), 8.17(m, 2H, Ph), 8.12 (s, 2H, Ph), 8.07 (d, 2H, *J* = 7.8 Hz, Ph), 7.70 (d, 1H, *J* = 1.8 Hz, im), 7.50 (d, 1H, *J* = 1.8 Hz, im), 5.32 (t, 8H, *J* = 7.8 Hz, CH_2_), 3.77 (s, 12H, *J* = 7.8 Hz, OMe), 3.53 (t, 8H, *J* = 7.8 Hz, CH_2_), 3.43 (s, 6H, NMe), 2.36–0.81 (m, 38H, alkyl groups in fluorene), −2.29 (s, 4H, NH); MALDI-TOF mass (dithranol), *m*/*z* 1587.75 (M + H)^+^, calcd for C_99_H_102_N_12_O_8_, 1586.79.

## 4. Conclusions

In conclusion, a porphyrin-fluorene conjugate connected by triple bonds **1** was successfully synthesized through a Pd-catalyzed coupling reaction. Polymer **1P**, self-assembled through zinc-imidazolyl complementary coordination, was successfully formed. The 2PA properties could be controlled by tuning the wavelength and absorption intensity of 1PA bands, which were altered by changing the interactions between the porphyrins by the introduction of fluorene or complementary coordination.
